# Differential Requirement for Protein Synthesis in Presynaptic Unmuting and Muting in Hippocampal Glutamate Terminals

**DOI:** 10.1371/journal.pone.0051930

**Published:** 2012-12-13

**Authors:** Devon C. Crawford, Xiaoping Jiang, Amanda Taylor, Krista L. Moulder, Steven Mennerick

**Affiliations:** 1 Graduate Program in Neuroscience, Washington University School of Medicine, St. Louis, Missouri, United States of America; 2 Department of Psychiatry, Washington University School of Medicine, St. Louis, Missouri, United States of America; 3 Department of Anatomy and Neurobiology, Washington University School of Medicine, St. Louis, Missouri, United States of America; 4 Taylor Family Institute for Innovative Psychiatric Research, Washington University School of Medicine, St. Louis, Missouri, United States of America; The Ohio State University, United States of America

## Abstract

Synaptic function and plasticity are crucial for information processing within the nervous system. In glutamatergic hippocampal neurons, presynaptic function is silenced, or muted, after strong or prolonged depolarization. This muting is neuroprotective, but the underlying mechanisms responsible for muting and its reversal, unmuting, remain to be clarified. Using cultured rat hippocampal neurons, we found that muting induction did not require protein synthesis; however, slow forms of unmuting that depend on protein kinase A (PKA), including reversal of depolarization-induced muting and forskolin-induced unmuting of basally mute synapses, required protein synthesis. In contrast, fast unmuting of basally mute synapses by phorbol esters was protein synthesis-independent. Further studies of recovery from depolarization-induced muting revealed that protein levels of Rim1 and Munc13-1, which mediate vesicle priming, correlated with the functional status of presynaptic terminals. Additionally, this form of unmuting was prevented by both transcription and translation inhibitors, so proteins are likely synthesized *de novo* after removal of depolarization. Phosphorylated cyclic adenosine monophosphate response element-binding protein (pCREB), a nuclear transcription factor, was elevated after recovery from depolarization-induced muting, consistent with a model in which PKA-dependent mechanisms, possibly including pCREB-activated transcription, mediate slow unmuting. In summary, we found that protein synthesis was required for slower, PKA-dependent unmuting of presynaptic terminals, but it was not required for muting or a fast form of unmuting. These results clarify some of the molecular mechanisms responsible for synaptic plasticity in hippocampal neurons and emphasize the multiple mechanisms by which presynaptic function is modulated.

## Introduction

Synapses are important sites for malleability of nervous system function. The silencing, or muting, of presynaptic terminals is arguably one of the least understood forms of synaptic malleability [1]. Mute synapses represent a class of terminals that completely fail to release transmitter in response to stimulation. Under basal conditions, a small population of hippocampal glutamatergic synapses is mute [2]–[5]. These silent terminals represent a reservoir that can rapidly [6], [7] or slowly [2], [5], [8]–[10] be activated for participation in neurotransmission. Furthermore, the population of basally active glutamatergic synapses can be muted selectively by strong depolarization [4], [11], including hypoxia-induced depolarization [[4], [11], [12], by inhibitory G-protein activation [13], and by direct decreases in cyclic adenosine monophosphate (cAMP) signaling [9]. Following a strong depolarizing challenge, muted synapses return to their active state within 3–4 h through a cAMP/PKA-dependent process [4], [9].

The complete signaling cascades responsible for muting and unmuting of hippocampal glutamatergic synapses remain unclear [1]. For example, slower forms of unmuting occur through cAMP-dependent signaling [5], [8]–[10] while rapid unmuting can be induced by phorbol esters [6]. These different time courses implicate disparate signaling cascades in unmuting. Relatively slow muting and unmuting could require protein synthesis or protein degradation. Proteasome activity is required for muting [14], suggesting a role for degradation. On the other hand, protein synthesis is required for some forms of persistent synaptic depression [15]–[17], so protein synthesis remains a plausible contributor to muting. Protein synthesis is also required for synaptic potentiation [18]–[20]; therefore, protein synthesis could also contribute to unmuting.

The present work, therefore, investigates the role of protein synthesis in presynaptic muting and unmuting in cultured hippocampal glutamatergic neurons. We found that muting after strong depolarization did not require protein synthesis. After depolarization was removed, the recovery of synaptic function and levels of the candidate unmuting protein Rim1 required both protein synthesis and PKA signaling. Levels of phosphorylated nuclear transcription factor CREB were elevated during the recovery period in a PKA-dependent manner, suggesting that CREB could be one intermediary through which PKA directs protein synthesis during the recovery period. Unmuting of basally mute terminals by phorbol esters did not require protein synthesis, however. We conclude, therefore, that protein synthesis is required for PKA-dependent unmuting of presynaptic terminals in glutamatergic hippocampal neurons, but muting of active terminals and unmuting by phorbol esters do not require protein synthesis.

## Methods

### Hippocampal Cell Culture

All procedures were carried out in accordance with the *Guide for the Care and Use of Laboratory Animals* published by the United States National Institutes of Health and were approved by the Washington University School of Medicine Animal Studies Committee. Primary hippocampal neuron cultures were prepared as previously described [21]. Hippocampi were removed from postnatal day 0–3 rat pups and incubated in 1 mg/ml papain. Cells were mechanically dissociated and plated as either “mass” cultures (∼650 cells/mm^2^ on a uniform layer of collagen) or “microisland” cultures (∼25 cells/mm^2^ on stamped microdots of collagen). Plating medium consisted of Eagle’s medium (Invitrogen) supplemented with heat-inactivated horse serum (5%), fetal bovine serum (5%), 17 mM glucose, 400 µM glutamine, 50 µg/ml streptomycin, and 50 U/ml penicillin. Cultures were maintained at 37°C in a humidified incubator under controlled atmospheric conditions (5% CO_2_/95% air). Cytosine arabinoside (6.7 µM) was added at 3–4 days *in vitro* (DIV) after plating to inhibit cell division. At d *in vitro* (DIV) 1 (mass cultures) or DIV 4–5 (microisland cultures), a medium exchange was performed with Neurobasal medium (Invitrogen) plus B27 supplement. All experiments were conducted on neurons 10–14 DIV.

To depolarize neurons strongly during muting induction, we added 30 mM KCl to the culture medium, which depolarizes the membrane potential to -20 to -30 mV [22], [23]. Ionotropic glutamate receptor blockers D-APV (25–50 µM) and NBQX (1 µM) were added to prevent toxicity and NMDA receptor-dependent plasticity. Controls were sibling cultures that received NaCl instead of KCl as a non-depolarizing osmotic control. For recovery, cultures were switched to fresh co-culture-conditioned medium or Neurobasal medium with B27 supplement for 3–4 h. For cycloheximide, KT5720 (EMD Millipore), and actinomycin D treatments, cultures were pretreated with each drug for 30–120 min immediately prior to manipulations described in the results. For cell death assays, cultures were treated with two doses of 50 µM C6-ceramide 5 h apart in the absence or presence of 1 µM cycloheximide.

### Electrophysiology

All electrophysiology experiments utilized microisland cultures. Sibling cultures plated on the same day from the same litter were always used to control for inter-plating variability. Whole-cell recordings were performed using pClamp version 9 or 10 software (Molecular Devices), an Axopatch 1D or Multiclamp 700B amplifier (Molecular Devices), and a Digidata 1322 acquisition board (Molecular Devices). Electrodes were 3–6 MΩ resistance, and access resistance was compensated 85–100%. Before recording, the culture medium was typically exchanged for recording solution (pH = 7.25) containing the following: 138 mM NaCl, 4 mM KCl, 2 mM CaCl_2_, 1 mM MgCl_2_, 10 mM glucose, 10 mM HEPES, and 25 µM D-APV. For conditions in which cells were previously treated with cycloheximide, 5 µg/ml cycloheximide was sometimes added to the recording saline to prevent the recurrence of protein synthesis during the recording session. Acute applications of cycloheximide did not have any effect on autaptic EPSC amplitudes (saline: -23.7±5.3 nA; saline+cycloheximide: -23.8±5.3 nA; *p* = 0.57, Student’s paired *t* test). The whole-cell pipette solution (pH = 7.25) contained the following: 140 mM K-gluconate, 500 µM CaCl_2_, 5 mM EGTA, and 10 mM HEPES. Neurons were stimulated by voltage pulses from −70 mV to 0 mV for 1.5 ms to evoke transmitter release and were recorded up to 1 h after switch to recording saline.

### Immunoblotting

Immunoblotting was performed as previously described [14]. Briefly, cultured neurons from four mass cultures per condition were separately collected for each experiment to account for dish-to-dish variability in cell number. Each dish of neurons was washed twice in phosphate-buffered saline (PBS) and lysed in 1x reducing Laemmli’s sample buffer plus protease inhibitors (10 µg/ml leupeptin and 20 µg/ml aprotinin). Samples from these four dishes per condition were loaded into separate lanes for separation. Individual samples were separated under reducing conditions using 4–12% Bis-Tris or 3–8% Tris-acetate NuPAGE gels (Invitrogen) and transferred to nitrocellulose. The blots were then incubated in 3% non-fat dried milk (NFDM) dissolved in 20 mM Tris, 137 mM NaCl, and 0.1% Tween 20 at a pH of 7.6 (TTBS). Primary antibodies [Munc13–1 (Synaptic Systems) at 1∶1000, Rim1 (BD Biosciences) at 1∶1000] and horseradish peroxidase-conjugated secondary antibodies were diluted in 1% NFDM in TTBS. Lumigen-TMA6 (GE Healthcare) or SuperSignal West Pico Chemiluminescent Substrate (Pierce) were used to detect bands while the Kodak ImageStation 440CF was used to capture the bands digitally. Densitometry was performed with Kodak 1D Image Analysis software. After image capture, blots were stripped using Restore Western Blot Stripping Buffer (Pierce) and re-probed with tubulin antibody (1∶4000) as a control for total protein levels or with SV2 antibody (1∶1000; Developmental Studies Hybridoma Bank, University of Iowa) as a control for presynaptic protein levels.

### Immunocytochemistry and Fluorescent Imaging

For immunocytochemistry of vesicle priming proteins, mass cultures were fixed in 4% paraformaldehyde/0.02% gluteraldehyde for 10 min before application of Rim1 (1∶500) or Munc13–1 (1∶500) primary antibody, typically combined with vesicular glutamate transporter 1 (vGluT-1) primary antibody (Millipore Corporation; 1∶2000). Secondary antibody for Rim1 or Munc13–1 was Alexa 647-conjugated anti-mouse antibody at 1∶500 dilution, and vGluT-1 secondary antibody was Alexa 488-conjugated anti-guinea pig antibody (1∶500; Life Technologies). MAP2 (1∶2000; EMD Millipore), pCREB (1∶400; EMB Millipore), CREB (1∶300; EMD Millipore), and GABA (1∶500; Millipore Corporation) primary antibodies were applied in some experiments after 10 min fixation in 4% paraformaldehyde/0.025–0.2% glutaraldehyde. Secondary antibodies were Alexa 488-conjugated anti-guinea pig antibody (1∶500; Life Technologies), Alexa 647-conjugated anti-rabbit antibody (1∶500; Life Technologies), and Cy3-conjugated anti-mouse antibody (1∶500; EMD Millipore), as appropriate. Glass coverslips were mounted onto microscope slides using Fluoromount-G (Southern Biotechnology).

Dye loading of presynaptic terminals was performed as previously described [24]. Briefly, fixable FM1-43 (FM1-43FX; 10 µM; Molecular Probes), 45 mM KCl, and 1 µM NBQX (Tocris Biosciences) in extracellular recording saline were applied for 2 min to mass cultures plated on glass coverslips. After ∼5 s rinse with 500 µM Advasep-7 (CyDex) in extracellular saline, neurons were washed with extracellular saline for 10 min and then fixed for 10 min in 4% paraformaldehyde/0.2% glutaraldehyde in PBS. Cultures were washed 3X in PBS and incubated in blocking solution (4% normal goat serum/0.04% Triton X-100 in PBS) for 15 min. vGluT-1 primary antibody was applied at 1∶2000 for 3 hr. After washing with PBS (3X), neurons were incubated in Alexa 647-conjugated anti-guinea pig antibody for 45 min (1∶500; Millipore Corporation), washed again in PBS (3X), and mounted onto microscope slides.

Fluorescent images were acquired using a Nikon C1 scanning confocal laser and 60x objective (1.4 numerical aperture) attached to an inverted Eclipse TE2000 microscope (Nikon). Images were acquired using EZ-C1 software (Nikon) by an observer naïve to experimental conditions. Representative fields were imaged in z-stack using alternating excitation by 488 nm, 543 nm, and/or 633 nm laser lines as appropriate while gain settings, pixel dwell, field of view size, and z-stack parameters were held constant throughout an experiment. Monochrome images were converted into 2D projected images for analysis using Metamorph Software (Universal Imaging).

### Data Analysis

All electrophysiology data were analyzed using Clampfit 9 or 10 (Molecular Devices) and Excel 2007 (Microsoft) software. At least 3 EPSCs were averaged and baseline-subtracted to measure autaptic EPSC amplitudes from individual neurons. For cell death assays, ten fields of neurons were counted with phase-contrast optics and a 20x objective by an observer naïve to conditions before ceramide treatment and 24 h after treatment. Cell death was quantified by calculating the percentage of cell loss after treatment compared to before treatment in the same dish. Confocal images were analyzed with Metamorph software by an observer naïve to the experimental conditions. For synaptic Rim1 and Munc13–1 quantification, regions were defined by vGluT-1 immunostaining, Rim1 or Munc13–1 integrated intensity was measured, and the values for all measured Rim1 or Munc13–1 puncta within a field were averaged for 5 fields per experiment. For pCREB and CREB experiments, average intensity was measured from 100 pixel square regions placed within the immunostained area. The percentage of active synapses was quantified as the percentage of vGluT-1-defined regions containing at least 10 thresholded pixels in the FM1-43FX image for 5 fields per experiment, as described in [13]. Graphs were created using SigmaPlot software (SPSS), and all quantifications are presented in the text and figures as mean ± SEM. Paired and unpaired Student’s *t* tests, with Bonferroni correction for multiple comparisons where appropriate, were used to determine if statistically significant differences were present unless otherwise stated (corrected *p*<0.05). For Rim1 immunostaining and Western blot experiments, one-way ANOVA followed by Newman-Keuls post hoc tests (*p*<0.05) were used.

### Materials

All reagents are from Sigma unless otherwise indicated.

**Figure 1:**
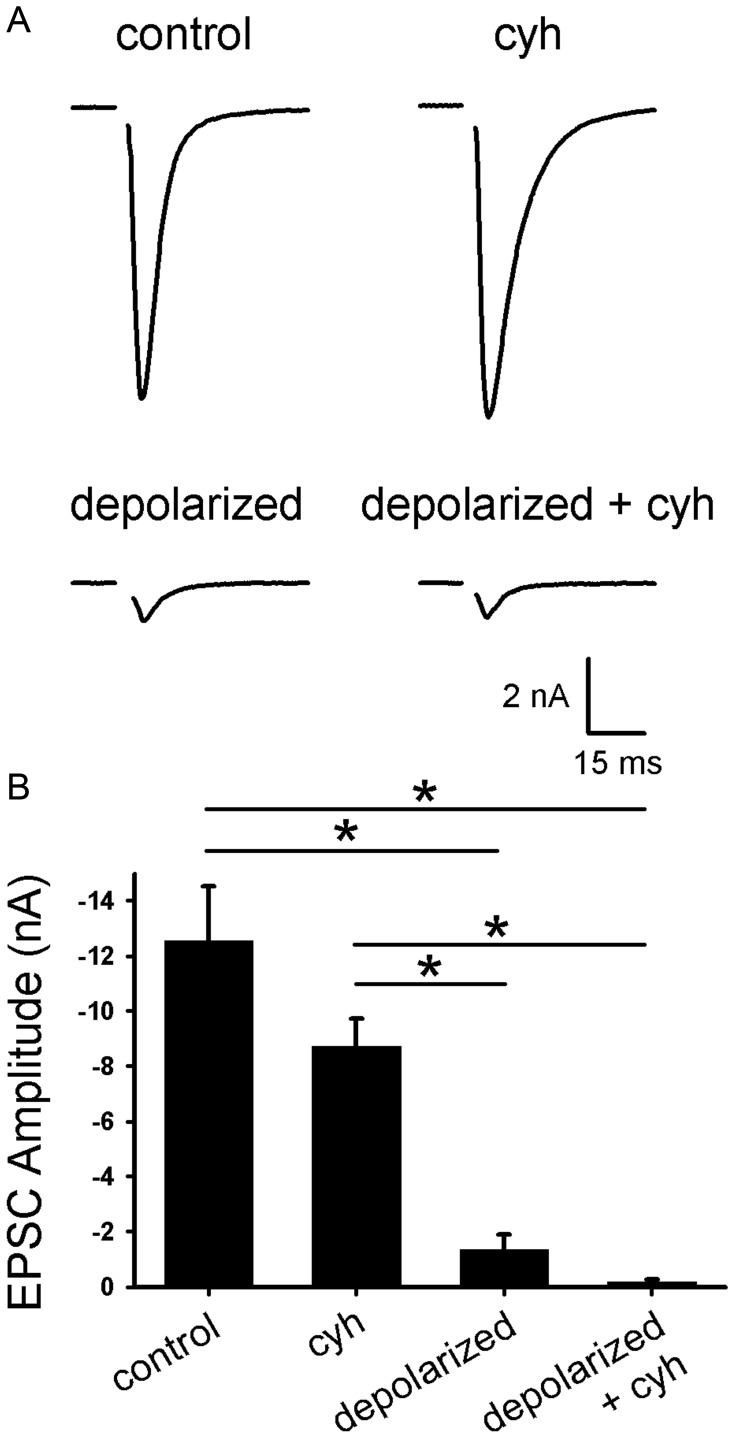
Protein synthesis is not required for depolarization-induced muting. A. Representative autaptic EPSCs from hippocampal neurons after 16 h 30 mM NaCl (control) or 30 mM KCl (depolarized) with or without 1 µg/ml cycloheximide 30 min pretreatment and co-incubation (cyh). **B.** Summary of EPSC amplitudes from neurons treated as in panel A (*n* = 10 neurons). *p<0.05, Bonferroni corrected Student’s unpaired *t* test.

## Results

### Protein Synthesis is not Required for Depolarization-induced Muting

Although many persistent forms of synaptic plasticity, including long-term depression [15]–[17], require protein synthesis, it is unknown whether protein synthesis plays a role in presynaptic muting induction. To test this, we applied cycloheximide (1–5 µg/ml), a rapidly reversible protein synthesis inhibitor that blocks translation elongation, during our induction protocol for presynaptic muting in hippocampal neurons. We focused on a prolonged, supraphysiological depolarization challenge (16 h; [4]) to produce strong synaptic depression and to invoke any late-phase components of depression most likely to require protein synthesis. We used autaptic excitatory postsynaptic current (EPSC) amplitudes to measure an estimate of presynaptic vesicle release; postsynaptic receptor expression is unaltered by this muting induction protocol while estimates of the readily releasable vesicle pool are strongly depressed [4], [11]. Evoked EPSC amplitudes were strongly depressed after 16 h depolarization (Fig. 1), as seen previously [4]. Cycloheximide co-incubation, however, did not alter the baseline EPSC amplitude or prevent the decreased amplitude after depolarization (Fig. 1). As a positive control for protein synthesis inhibition by cycloheximide, we examined ceramide-induced apoptosis in hippocampal neurons. Ceramide (2 doses of 50 µM) increased hippocampal neuron death over a 24 h period (untreated: 26±8% death; ceramide: 55±10% death; *p* = 0.0008, Bonferroni corrected; *n* = 5). This neuron loss was prevented by 1 µM cycloheximide (cycloheximide: 33±10% death; cycloheximide+ceramide: 25±10% death; *p* = 0.74 before Bonferroni correction; *n* = 4). Taken together, these data suggest that protein synthesis is not required for depolarization-induced presynaptic muting.

**Figure 2:**
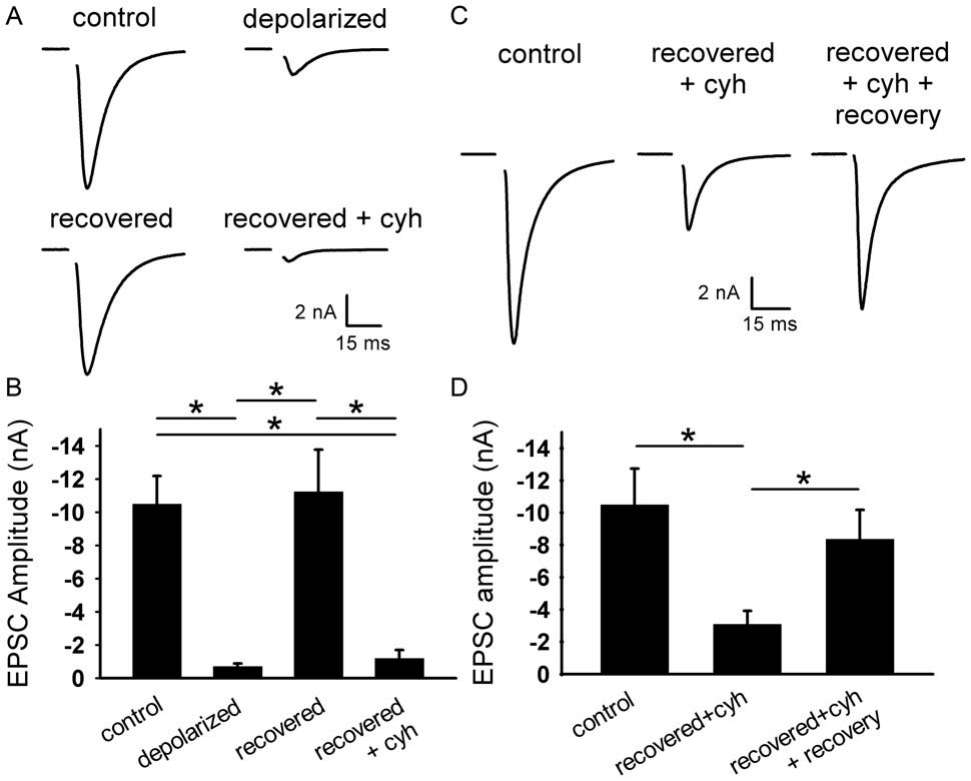
Protein synthesis is required for functional recovery from depolarization-induced muting. **A.** Representative autaptic EPSCs from hippocampal neurons after 16 h 30 mM NaCl (control), 16 h 30 mM KCl (depolarized), or 16 h 30 mM KCl followed by 3 h recovery in fresh medium with (recovered+cyh) or without (recovered) 1–5 µg/ml cycloheximide. Cycloheximide was applied 0.5–2 h prior to and during recovery. **B.** Summary of EPSC amplitudes from neurons treated as in panel A (*n* = 14 neurons). **C.** Representative autaptic EPSCs from hippocampal neurons after 16 h 30 mM NaCl (control), 16 h 30 mM KCl followed by 3 h recovery in fresh medium with 5 µg/ml cycloheximide (recovered+cyh), or 16 h 30 mM KCl followed by 3 h recovery in fresh medium with 5 µg/ml cycloheximide followed by an additional 3 h recovery in fresh medium without cycloheximide (recovered+cyh+recovery). **D.** Summary of autaptic EPSC amplitudes from neurons treated as in panel C (*n* = 25 neurons). *p<0.05, Bonferroni corrected Student’s unpaired *t* test.

### Protein Synthesis is Required for Recovery from Depolarization-induced Muting

Increased muting correlates with a proteasome-dependent reduction in the levels of priming proteins Rim1 and Munc13–1 [14]. It is possible, therefore, that recovery from muting requires synthesis of these proteins for presynaptic terminals to regain function. Protein synthesis is required for PKA-dependent unmuting of basally mute synapses [7], [8], but unmuting of pathophysiologically muted synapses has not been explored. Recovery from synaptic depression triggered by strong, prolonged depolarization requires 3–4 h [4]; therefore, recovery from muting is slow enough to engage protein synthesis pathways.

**Figure 3:**
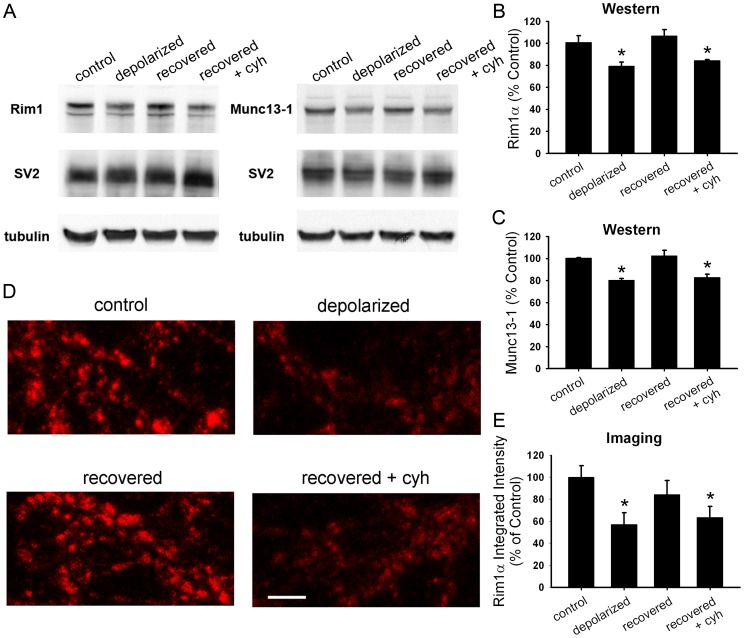
Synthesis is required for protein recovery from depolarization-induced muting. A. Western blot analysis of whole-cell lysates from hippocampal mass cultures treated with 16 h 30 mM NaCl (control), 16 h 30 mM KCl (depolarized), or 16 h 30 mM KCl followed by 3 h recovery in fresh medium with (recovered+cyh) or without (recovered) 1 µg/ml cycloheximide. Cycloheximide was applied 0.5 h prior to and during recovery. **B.** Summary of Rim1 levels from Western blots as shown in A (*n* = 3). Rim1 protein levels for each condition were normalized to SV2 and control treatment. **C.** Summary of Munc13–1 levels from Western blots as shown in A (*n* = 3). Munc13–1 protein levels for each condition were normalized to SV2 and control treatment. **D.** Representative images of Rim1 immunostaining in mass cultures after treatments as described in panel A. Scale bar represents 2 µm. **E.** Quantification of Rim1 immunostaining at vGluT-1-positive synapses (not shown; *n* = 15 fields). Integrated intensity values were normalized to the average control value for a given experiment. *p<0.05, Newman-Keuls post hoc test vs. control after one-way ANOVA.

To test the hypothesis that protein synthesis is required for recovery from muting, we blocked protein synthesis with cycloheximide during a 3 h recovery period following the 16 h depolarization challenge. Cycloheximide prevented the recovery of the EPSC amplitude from depolarization-induced muting (Fig. 2A and 2B). When cycloheximide was removed for an additional 3 hr, however, EPSC amplitudes recovered (Fig. 2C), demonstrating that cycloheximide did not permanently alter presynaptic function. This suggests that recovery from presynaptic muting requires protein synthesis.

**Figure 4:**
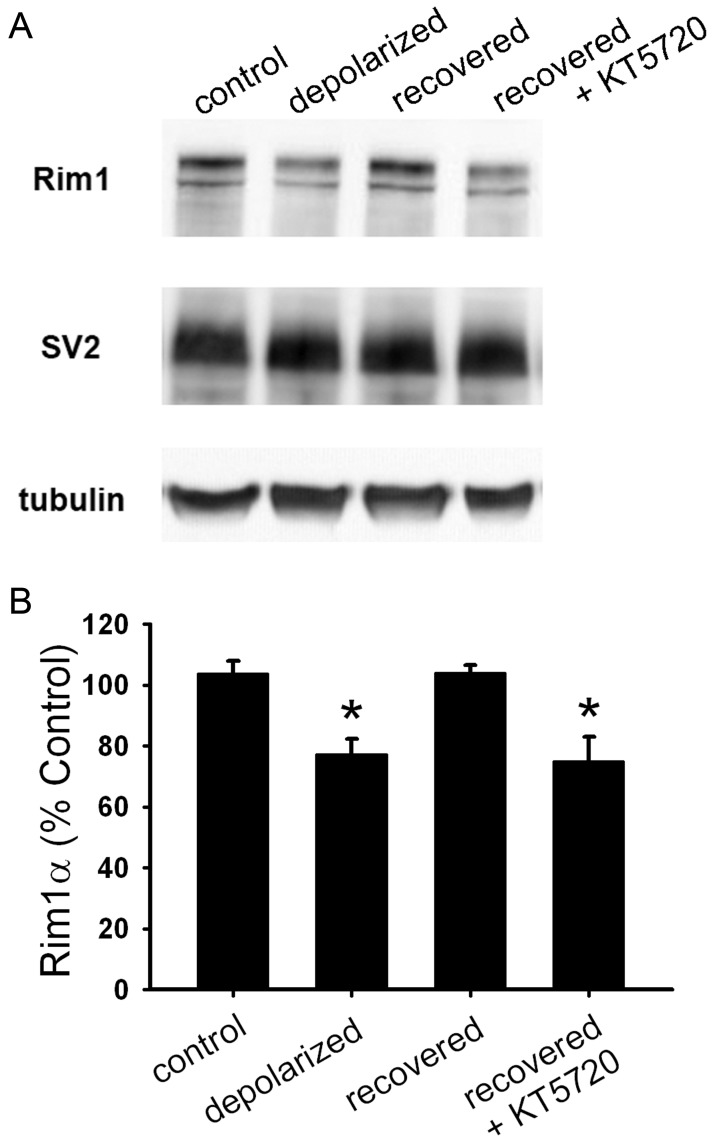
PKA signaling is required for recovery of Rim1 levels after depolarization-induced muting. A. Western blot analysis of whole-cell lysates from hippocampal mass cultures treated with 16 h 30 mM NaCl (control), 16 h 30 mM KCl (depolarized), or 16 h 30 mM KCl followed by 3 h recovery in fresh medium with (recovered+KT5720) or without (recovered) 2 µM KT5720. KT5720 was applied 0.5 h prior to and during recovery. **B.** Summary of Rim1 levels from Western blots as shown in A (*n* = 3). Rim1 protein levels for each condition were normalized to SV2 and control treatment. *p<0.05, Newman-Keuls post hoc test vs. control after one-way ANOVA.

**Figure 5:**
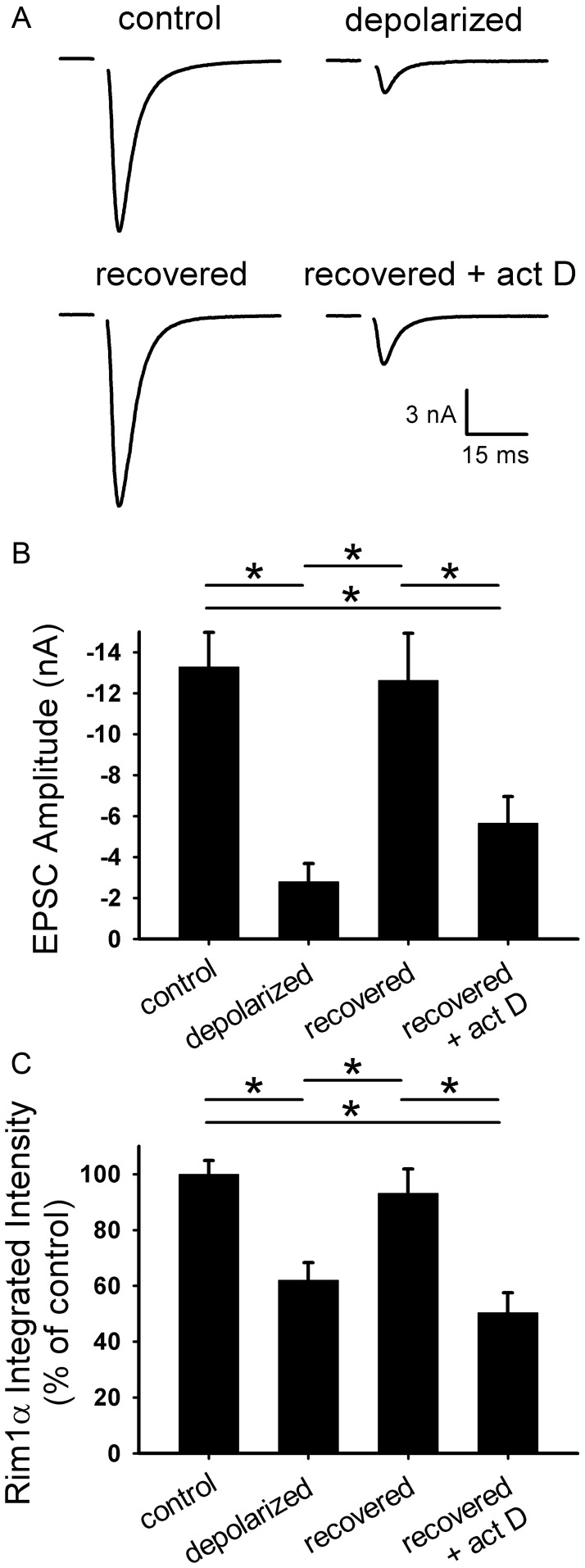
Transcription is required for functional recovery from depolarization-induced muting. A. Representative autaptic EPSCs from hippocampal neurons after 16 h 30 mM NaCl (control), 16 h 30 mM KCl (depolarized), or 16 h 30 mM KCl followed by 3 h recovery in fresh medium with (recovered+act D) or without (recovered) 200 ng/ml actinomycin D. Actinomycin D was applied 0.5 h prior to and during recovery. **B.** Summary of EPSC amplitudes from neurons treated as in panel A (*n* = 32 neurons). **C.** Quantification of Rim1 immunostaining at vGluT-1-positive synapses (not shown; *n* = 15 fields). Integrated intensity values were normalized to the average control value for a given experiment. *p<0.05, Bonferroni corrected Student’s one-tailed unpaired *t* test.

Because loss of vesicle priming proteins Rim1 and Munc13–1, but not loss of other presynaptic proteins, is associated with muting [14], recovery of Rim1 and Munc13–1 levels may be responsible for recovery of presynaptic function. To test if protein levels recover in parallel with functional recovery, we measured Rim1 and Munc13–1 levels after recovery with Western blot. Both proteins decreased after depolarization but recovered to control levels within 3 h of switch to fresh, non-depolarizing medium (Fig. 3A–C). In the presence of cycloheximide, protein levels did not recover (Fig. 3A–C). These results were confirmed by measuring the integrated intensity of Rim1 immunostaining at glutamatergic synapses. Immunofluorescence intensity of Rim1 puncta decreased after 16 h depolarization and recovered within 3 h of switch to fresh medium (Fig. 3D and 3E). Cycloheximide prevented the recovery in Rim1 immunofluorescence intensity (Fig. 3D and 3E), suggesting that Rim1 levels, like synaptic function, fail to recover in the presence of protein synthesis inhibitors. We also measured Munc13–1 immunofluorescence intensity at glutamatergic synapses treated with cycloheximide during the recovery period. As expected, Munc13–1 levels were depressed after recovery from depolarization in the presence of cycloheximide when compared to levels in non-depolarized cells (70.2±6.9% of control; p = 0.02, Bonferroni corrected; *n* = 20 fields). As with synaptic function, further recovery in the absence of cycloheximide restored Munc13–1 levels (118.5±9.7% of control; p = 0.44 vs. control, p<0.001 vs. recovery+cycloheximide, Bonferroni corrected; *n* = 20 fields). Together, these data suggest that protein synthesis is important for the recovery of priming protein levels after depolarization has ceased. These results also support the previous observation that Rim1 and Munc13–1 presynaptic functions are linked with each other [25]–[27] and that their levels, unlike those of other proteins, correlate with the percentage of non-mute presynaptic terminals [14].

**Figure 6:**
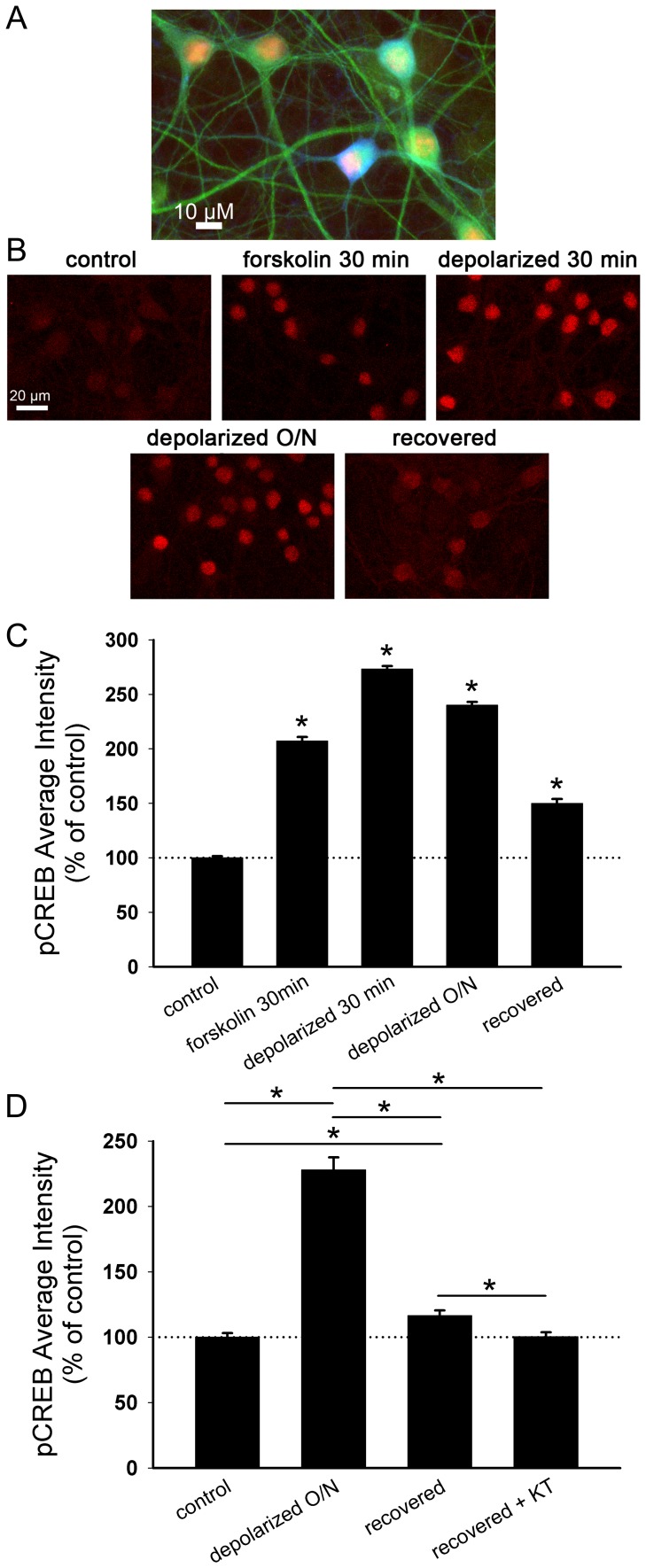
Nuclear phosphorylated CREB (pCREB) levels remain elevated after recovery from depolarization-induced muting. A. Merged image of pCREB (red), GABA (blue), and MAP2 (green) immunofluorescence in a mass hippocampal culture. **B.** pCREB immunostaining after the following treatments: 30 min DMSO (control), 30 min 50 µM forskolin (forskolin 30 min), 30 min 30 mM KCl (depolarized 30 min), 16 h 30 mM KCl (depolarized O/N), or 16 h 30 mM KCl followed by 3 h in fresh medium (recovered). **C.** Quantification of pCREB intensity in GABA-negative nuclei after treatments as described in panel A (*n* = 327–586 neurons). Intensity values were normalized to the average control value for each experiment. *p<0.05, Student’s unpaired *t* test vs. control after Bonferroni correction. **D.** Quantification of pCREB intensity in GABA-negative nuclei after 16 h 30 mM NaCl (control), 16 h 30 mM KCl (depolarized O/N), or 16 h 30 mM KCl followed by 3 h recovery in fresh medium with (recovered+KT) or without (recovered) 2 µM KT5720 (*n* = 45–145 neurons). KT5720 was applied 0.5 h prior to and during recovery. Intensity values were normalized to the average control value for each experiment. *p<0.05, Bonferroni corrected Student’s unpaired *t* test.

### PKA Signaling is Required for Recovery from Depolarization-induced Muting

Previous results suggest that cAMP- and PKA-dependent pathways modulate presynaptic muting and unmuting over the course of hours [1], [9], [13]. Additionally, inhibition of PKA signaling with KT5720 prevents recovery of presynaptic function after muting-inducing depolarization has been removed [9]. It is unclear, however, whether the recovery of priming protein levels also requires PKA signaling. We measured Rim1 levels using Western blot after 16 h depolarization followed by 3 h recovery in the presence of 2 µM KT5720. Levels of Rim1 remained depressed when cells were incubated in KT5720 during the recovery period (Fig. 4), suggesting that PKA signaling is required for recovery of Rim1 levels from muting.

Because both synaptic function [9] and protein levels (Fig. 4) require PKA to recover, PKA-dependent synthesis of presynaptic priming proteins may be necessary for the recovery of presynaptic function after a depolarizing stimulus is removed. We hypothesized that PKA signaling upregulates transcription during recovery. Consistent with a role for *de novo* transcription, rather than local translation, we found that applying the transcription inhibitor actinomycin D (200 ng/ml) during recovery also blocked functional recovery from muting (Fig. 5A and 5B). Similarly, we found that actinomycin D prevented recovery of Rim1 levels at excitatory synapses, as measured with immunofluorescence (Fig. 5C). These results suggest that transcription, and not just translation, are required for recovery of presynaptic protein levels and function.

**Figure 7:**
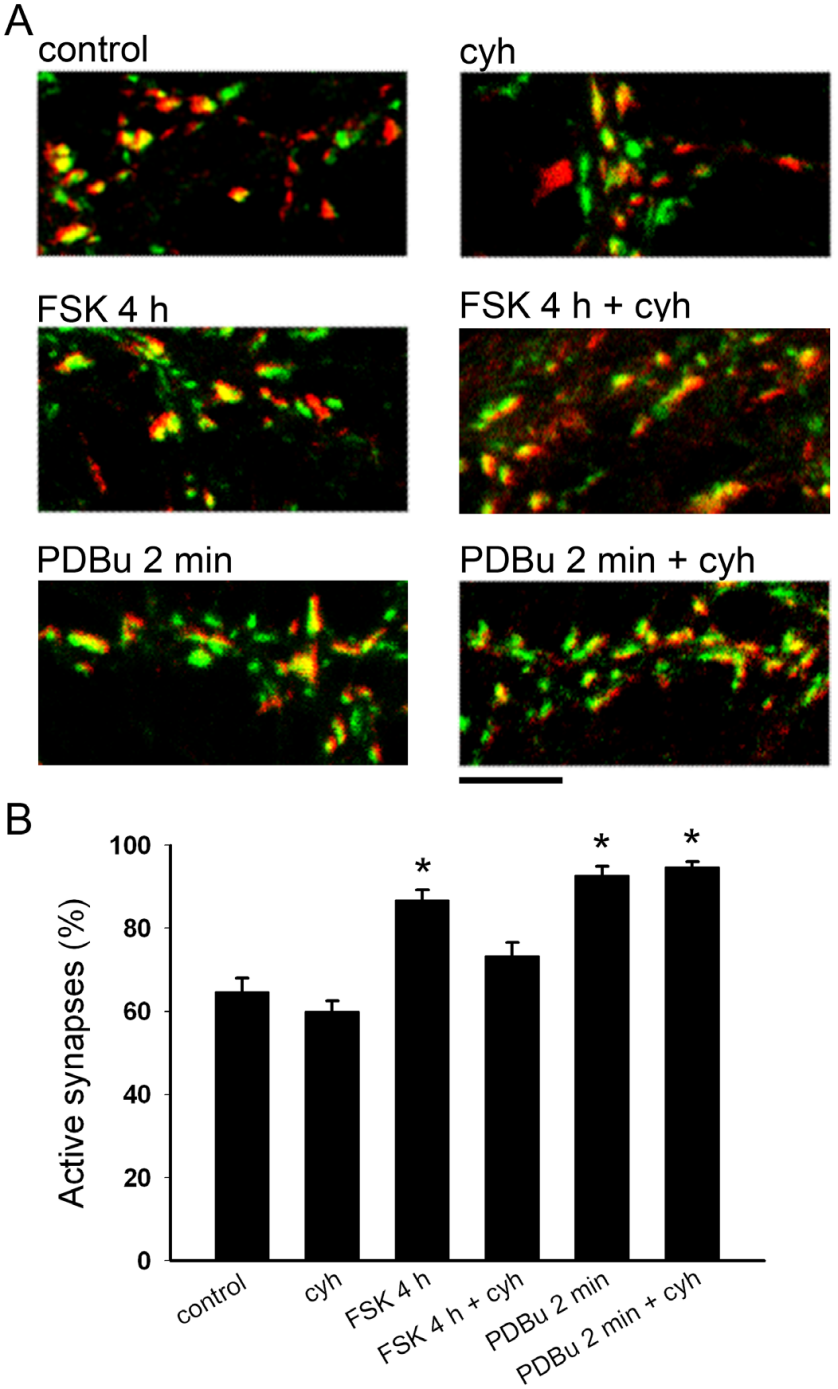
Unmuting of basally mute synapses by forskolin, but not PDBu, requires protein synthesis. **A.** Representative FM1-43FX (green) and vGluT-1 (red) merged images after 4 h DMSO (control), 4 hr DMSO plus 1 µg/ml cycloheximide (cyh), 4 h 50 µM forskolin (FSK 4 h), 4 h 50 µM forskolin plus 1 µg/ml cycloheximide (FSK 4 h+cyh), 2 min 1 µM PDBu (PDBu 2 min), or 2 min 1 µM PDBu plus 1 µg/ml cycloheximide (PDBu 2 min+cyh) treatment. Cycloheximide was applied 0.5 h prior to and during treatments. Scale bar represents 5 µm. **B.** Quantification of the percentage of vGluT-1-defined synapses labeled with FM1-43FX (active synapses) after treatments described in panel A (*n* = 20 fields). *p<0.05, Bonferroni corrected Student’s unpaired *t* test vs. control.

If cAMP/PKA-dependent protein synthesis is responsible for unmuting, we would expect markers of PKA-dependent protein synthesis to correlate with the recovery from depolarization-induced muting. As a proxy for cAMP/PKA-dependent protein transcription, we measured levels of pCREB immunostaining in the nuclei of glutamatergic neurons during strong depolarization and during the recovery period after depolarization was removed (Fig. 6A). pCREB average intensity levels were elevated throughout depolarization and remained higher than in control-treated neurons after 3 h recovery (Fig. 6B and 6C). We also examined pCREB levels in neurons treated with 30 min 50 µM forskolin, which stimulates adenylyl cyclase-dependent production of cAMP. Forskolin-treated neurons clearly exceeded baseline levels of pCREB, but pCREB levels were not as strong as in depolarized neurons (Fig. 6B and 6C). KT5720 application during recovery from depolarization prevented the increased pCREB levels, even though non-depolarized neurons were unaffected by KT5720 (Fig. 6D). Levels of total nuclear CREB protein were also elevated after depolarization (37.8±4.6% increase; *p*<0.001, Bonferroni corrected; *n* = 125 neurons), potentially contributing to the depolarization-induced increase in pCREB; however, total CREB levels after recovery with or without KT5720 were not significantly different from the non-depolarized control condition (recovered: 4.9±3.5% increase; recovered+KT5720∶6.8±3.2% increase; *p*>0.05, Bonferroni corrected; *n* = 125 neurons). This pattern suggests that elevated PKA activity, rather than activity of other kinases or changes in total protein levels, is responsible for increased CREB phosphorylation during the recovery period.

Although we used nuclear pCREB accumulation solely as a correlative marker of PKA activity, the increased pCREB in recovering neurons provides a possible substrate for cAMP/PKA-dependent transcriptional regulation of recovery and the correlated net protein level increases. These data alone do not exclude the possibility that elevated PKA activity during depolarization sows the seeds of recovery (Fig. 6C, D), but our previous work has demonstrated that PKA inhibitors KT5720 and Rp-cAMPS applied only during the recovery period prevent unmuting [9], suggesting that PKA-dependent processes are recruited after removal of depolarization. Together with the present results, this strengthens the evidence that cAMP-dependent transcription and subsequent protein translation after removal of depolarization are important for unmuting glutamatergic presynaptic terminals.

### Basally Mute Synapses are Unmuted by Protein Synthesis-dependent and –independent Mechanisms

A sizable proportion (∼25–50%) of hippocampal glutamatergic synapses are mute prior to experimental manipulations [3]–[5]. We and others have previously shown that cAMP-dependent pathways [5], [7]–[10], [28], [29] and phorbol esters [6], [9] unmute some of these basally mute synapses. The time courses required for these two unmuting treatments are remarkably different, with phorbol esters unmuting synapses after a couple minutes of treatment and forskolin application requiring minutes to hours. The faster effects of phorbol esters suggest that phorbol ester-induced unmuting may be independent of protein synthesis, so we compared the protein synthesis dependence of each of these treatments. We used presynaptic vesicle labeling with FM1-43FX dye rather than autaptic EPSCs to determine whether terminals are unmuted to avoid inadvertent intrusion of other presynaptic and postsynaptic modifications from forskolin and phorbol ester treatment [30]–[35]. As expected, both 4 h of forskolin (50 µM) and 2 min of phorbol 12,13-dibutyrate (PDBu; 1 µM) increased the percentage of glutamatergic terminals exhibiting FM1-43FX fluorescence after brief stimulation, which indicates an increase in the fraction of functional presynaptic terminals (Fig. 7). Cycloheximide co-incubation, however, prevented forskolin-induced unmuting but not phorbol ester-induced unmuting (Fig. 7). These results suggest that multiple molecular pathways unmute hippocampal glutamatergic presynaptic terminals, and the slower, PKA-dependent pathways require protein synthesis.

## Discussion

Here we present evidence that protein synthesis is involved in PKA-dependent unmuting of hippocampal glutamatergic presynaptic terminals. Muting in response to strong depolarization did not require protein synthesis. Recovery of synaptic function and vesicle priming protein levels after termination of depolarization, however, was prevented by translation and transcription inhibitors. PKA inhibition also hindered recovery of a candidate protein mediator and prevented the elevation of pCREB levels during the recovery period. Combined with our previous data suggesting that PKA activity is necessary for functional recovery [9], these results suggest that PKA-dependent protein synthesis may be important for recovery from muting. Levels of priming proteins Rim1 and Munc13–1 correlated with synaptic function, suggesting that these proteins are important for synapses to remain active. Unmuting of basally mute synapses by forskolin also required protein synthesis, although unmuting by phorbol esters did not. This suggests that unmuting via PKA-dependent pathways, whether recruited endogenously after removal of an excitatory stimulus or induced pharmacologically in non-depolarized cells, requires formation of new protein.

Many persistent forms of synaptic plasticity require protein synthesis. For example, Hebbian late-phase long-term potentiation in the hippocampus requires protein synthesis [36]–[38]. Similarly, evidence suggests that protein synthesis contributes to hippocampal long-term depression [36], [39]. Homeostatic forms of plasticity, like synaptic scaling of AMPA receptors during prolonged activity deprivation or enhancement, also require the synthesis of new protein [40]–[42]. Interestingly, many of these forms of plasticity are mediated by postsynaptic receptor changes and require local, dendritic translation into protein but not DNA transcription. In contrast, presynaptic unmuting after the removal of depolarization in our study required both transcription and translation, suggesting that local protein synthesis does not play a strong role. This clarifies ambiguity in previous work, which tested for a role of translation, but not transcription, in PKA-dependent activation of dormant neurotransmitter release sites [7], [8]. Unmuting could involve activation of the transcription factor CREB or other PKA-dependent transcription mechanisms. In our study, CREB phosphorylation was elevated during the recovery period in a PKA-dependent manner. PKA-dependent CREB phosphorylation is involved in protein synthesis under many contexts [43]–[45], so it is plausible that nuclear CREB links PKA activity to protein synthesis during unmuting. Future work could explicitly test the role of pCREB-dependent transcription, for example by inhibiting CREB with dominant-negative genetic manipulations [46]–[50].

In this study, levels of the presynaptic proteins Rim1 and Munc13–1 correlated with synaptic function. A causal role for Rim1 and Munc13–1 degradation in muting has previously been suggested [14]. In the current study, Rim1 and Munc13–1 levels decreased after muting induction, returned to baseline during the recovery period, and remained at low levels when neurons recovered from depolarization in the presence of protein synthesis or PKA inhibitors. Rim1 and its molecular partner Munc13–1 are important for vesicle priming [25], [51]–[54], which is consistent with the priming deficit induced by muting [11], [55], [56]. Prior work suggests that Rim1 and Munc13–1 levels are selectively decreased during muting since levels of several other presynaptic proteins remain stable during muting induction [14]. Rim1α overexpression prevents muting and preserves Munc13–1 levels [14], and forskolin treatment that unmutes basally mute terminals increases Rim1 protein [9]. Altogether, this suggests that Rim1, and potentially Munc13–1, synthesis participates in PKA-dependent unmuting.

Protein synthesis was not required for muting, but evidence suggesting that proteasome activity is required for loss of Rim1 and Munc13–1 during muting further supports our hypothesis that levels of these proteins mediate muting status [14]. Although depolarization increased pCREB levels, suggesting that protein transcription is recruited during muting induction, increased proteasome activity caused by depolarization [14] may induce protein degradation that overcomes protein synthesis and causes a net decrease in protein levels. We hypothesize that return to normal levels of proteasome activity after removal of depolarization unmasks effects of protein synthesis, allowing for a net increase in protein levels during recovery. Rim1α is a PKA substrate [57], however, so effects on synaptic function by additional posttranslational modifications to Rim1α are not ruled out by our studies.

Additional complexities are likely to cloud the simple hypothesis that Rim1 and Munc13–1 levels explain muting and unmuting. The strong synaptic phenotype with mild reductions in priming protein levels is not straightforwardly consistent with genetic deletion studies showing mild functional deficits. Homozygous null Rim1 animals exhibit reduced, but not extinguished, synaptic functionality [26], [53], possibly suggesting a degree of redundancy among Rim isoforms [58]. Munc13–1 and Munc13–2 double knockouts, in contrast, exhibit negligible synaptic function [56], [59], suggesting that Munc13 levels may be more important than Rim1 levels in determining synaptic functional status. Because both Rim1 and Munc13–1 levels correlate with the percentage of non-mute synapses, loss of both proteins simultaneously could mediate the muting phenotype. This does not, however, rule out other protein mediators or mechanisms that could be playing a role. Additionally, we are not aware of studies directly linking CREB phosphorylation with Rim1 or Munc13–1 transcription, so other PKA-dependent regulators besides CREB or other intermediate regulators downstream of CREB may be activated during unmuting. Future work should clarify the pathway responsible for unmuting-associated protein synthesis.

Interestingly, unmuting was induced via both protein synthesis-dependent and -independent mechanisms. Protein synthesis-dependent unmuting from baseline or following depolarization-induced muting both exhibited PKA dependence. Unmuting of basally mute synapses by PDBu, which we found was protein synthesis-independent, does not require PKC activity [6]. Phorbol ester unmuting, therefore, likely depends on direct interactions with Munc13–1 [6], [60]–[62]. One obvious difference between PKA-dependent and phorbol ester-dependent unmuting is the time scale required. A mere 2 min of PDBu application unmutes basally mute synapses, but 2 min of forskolin has shown no effect on the number of mute terminals in prior studies [6]. To unmute synapses, PKA-dependent pathways required hours, not minutes, which can be explained by the involvement of transcription and translation.

Some previous studies have shown slow cAMP- or PKA-dependent awakening of basally dormant synapses [5], [7]–[10], [28], [29], but our study additionally clarifies mechanisms involved in homeostatic unmuting after removal of an excitatory stimulus. Although our work implies that unmuting of depolarization-muted terminals and basally mute terminals utilize the same signaling cascades, prior work in genetic models argues that the signaling cascades are more complex. Neurons doubly deficient in adenylyl cyclase 1 and 8 fail to recover from depolarization-induced muting, attributable to the loss of adenylyl cyclase 8, but these terminals are awakened with forskolin activation [9]. This suggests that forskolin application recruits additional adenylyl cyclase isoforms than are endogenously recruited during recovery from muting. Therefore, some downstream effectors may be differentially activated by forskolin unmuting and homeostatic unmuting. Unmuting after removal of depolarization may be triggered by sensitization of adenylyl cyclases by the prolonged inhibitory G-protein signaling activated during muting [13], [63], but other pathways may be involved in other forms of unmuting. These findings highlight the multiple mechanisms by which presynaptic unmuting can be manipulated and open the door to many questions about physiological roles of muting and unmuting. For example, it is conceivable that slower forms of muting and unmuting mediate homeostatic and Hebbian plasticity in response to network activity while faster unmuting may meet the demands of dynamic, moment-to-moment nervous system function.

In summary we have described a form of adaptive presynaptic muting induced by strong depolarization that did not require protein synthesis but that past work has shown depends on protein degradation. Recovery from this muting required both protein synthesis and PKA activity, suggesting that cAMP signaling controls presynaptic protein levels and neurotransmitter release. This slow form of unmuting contrasts with much faster unmuting by phorbol esters and emphasizes the multiple mechanisms controlling neurotransmission competency of presynaptic terminals. In addition, priming proteins Rim1 and Munc13–1 are viable candidate downstream targets for these changes. Our results contribute to our understanding of the downstream events controlling presynaptic functional status; however, many upstream signals in the cascade remain to be elucidated [1]. Because presynaptic muting is endogenously neuroprotective *in vitro* [12], clarifying the complete signaling cascades involved in muting and unmuting could aid development of therapies for conditions involving dysregulated glutamate signaling.
